# Flagellin sensing, signaling, and immune responses in plants

**DOI:** 10.1016/j.xplc.2025.101383

**Published:** 2025-05-20

**Authors:** Hyeonmin Ryu, Sejin Choi, Mengwei Cheng, Bon-Kyoung Koo, Eun Yu Kim, Ho-Seok Lee, Du-Hwa Lee

**Affiliations:** 1 Department of Biology, College of Sciences, Kyung Hee University, Seoul 02447, Korea; 2 Center for Genome Engineering, Institute for Basic Science, Daejeon 34126, Korea; 3 Division of Natural and Applied Sciences, Duke Kunshan University, Kunshan, Jiangsu 215300, China; 4 Environment Research Center, Duke Kunshan University, Kunshan, Jiangsu 215300, China; 5 Department of Chemistry, BOKU University, Muthgasse 18, 1190 Vienna, Austria

**Keywords:** flagellin sensing, FLS2 receptor, pattern-triggered immunity, PTI, pattern-recognition receptors, PRRs, microbe-associated molecular patterns, MAMPs, plant–pathogen interaction

## Abstract

The flagellin-sensing mechanism is one of the most extensively studied topics in plant defense systems. This widespread interest arises from the ability of flagellin to trigger robust and extensive responses, establishing it as a cornerstone for research into other defense mechanisms. Plants recognize bacterial flagellin epitopes through plasma-membrane-localized pattern-recognition receptors, initiating pattern-triggered immunity as the frontline defense against bacterial pathogens. In this review, we comprehensively summarize flagellin-sensing mechanisms and signal transduction pathways in plants. We compare the flagellin-sensing mechanisms of plants and mammals, focusing on epitope processing and recognition. We present detailed downstream signaling events, from receptor complex formation to transcriptional reprogramming. Furthermore, we highlight the evolutionary arms race between plants and bacteria and incorporate emerging insights into how flagellin-triggered responses are modulated by receptor networking, phytocytokines, and environmental factors. These findings suggest that flagellin-mediated immune responses are highly dynamic and context dependent. By synthesizing current knowledge and recent discoveries, this review provides updated perspectives on plant–microbe interactions and aims to inspire future research in plant immunity.

## Teaser

Plants detect bacterial flagellin through plasma-membrane-localized pattern-recognition receptors (PRRs), initiating pattern-triggered immunity (PTI) as an early defense response. This review provides a comprehensive overview of flagellin perception mechanisms, downstream signaling pathways, and plant–bacteria interactions. It also highlights recent discoveries on how plants modulate flagellin-mediated immunity and proposes a conceptual framework for understanding broader PRR-mediated PTI responses in plants.

## Introduction

**Figure 1:**
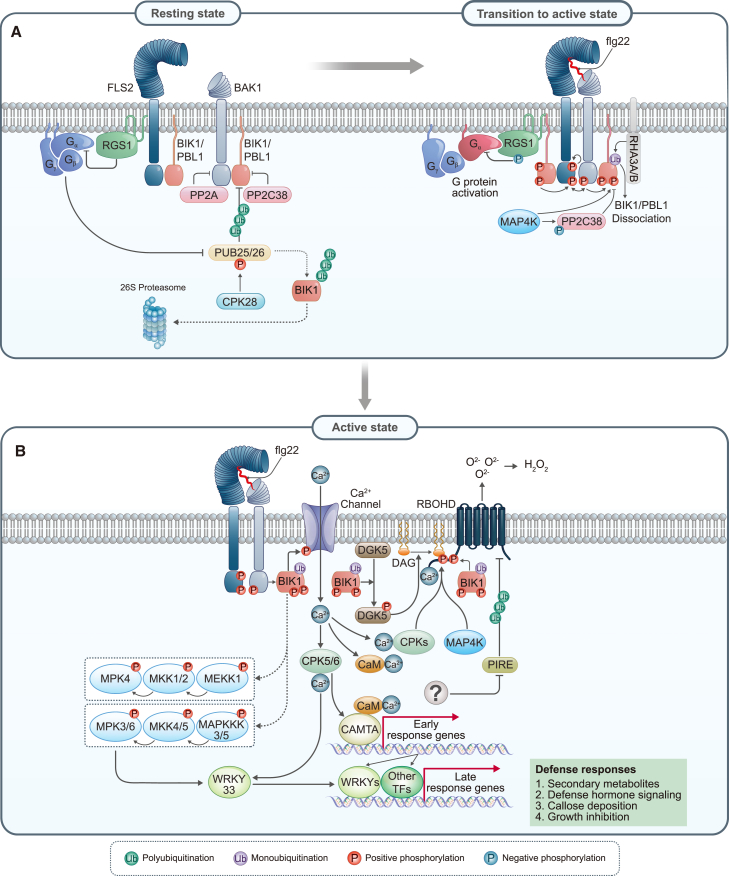
The overall signal-transduction pathway mediated by FLS2. **(A)** Resting state and initial activation of the FLS2 receptor complex. In the resting stage, BIK1 interacts with FLS2 and BAK1. BAK1 and BIK1 are negatively regulated by the protein phosphatases PP2A and PP2C38, respectively. PP2C38 is negatively regulated by phosphorylation mediated by MAP4K. BIK1 is polyubiquitinated by PUB25/26 and degraded by the 26S proteasome. Upon perception of flg22 by FLS2, BAK1 is recruited, and they cross-phosphorylate each other. The activated FLS2–BAK1 heterocomplex then transphosphorylates BIK1. Monoubiquitination of BIK1, mediated by RHA3/B, is required for its dissociation from the FLS2–BAK1 complex. **(B)** Active state and downstream signaling events of the FLS2 receptor complex. Activated BIK1 leads to the opening of Ca^2+^ channels, activating DGK5 and RBOHD through phosphorylation. RBOHD is positively regulated by CPKs in a Ca^2+^-dependent manner and negatively regulated by the E3 ubiquitin ligase PIRE. The MAPK kinase cascade is also activated, primarily in a BIK1-dependent manner, and subsequently activates WRKY TFs. Increased cytosolic Ca^2+^ is sensed by CaM and regulates early response genes through CAMTA TFs. Directly activated WRKYs, along with transcriptionally induced WRKYs and other TFs, result in significant transcriptomic changes. These changes contribute to the accumulation of defense-related secondary metabolites, activation of defense-hormone signaling, callose deposition, and growth inhibition.

At the frontline of plant–pathogen interactions, the recognition of microbial invaders serves as a critical step in the plant’s defense systems. At the forefront of this defense line lie pattern-recognition receptors (PRRs), which are localized on the plasma membrane (PM) to detect conserved molecular signatures known as microbe-associated molecular patterns (MAMPs) at the extracellular space, called the apoplast. During the ongoing evolutionary arms race between pathogens and host plants, plants have evolved to expand both the number and variety of receptors on their surfaces to effectively sense intricate molecular signals from pathogen challenges (Lee et al., 2021). Upon recognition of MAMPs by PRRs, plants initiate a cascade of defense responses termed pattern-triggered immunity (PTI), thereby fortifying host resistance against pathogen invasion (Figure 1).

Among these recognized MAMPs, flagellin, a ubiquitous basal component of bacterial flagella, holds particular importance across multiple kingdoms of life owing to its widespread presence in diverse pathogenic bacteria. Flagellin is therefore a prime target for host detection, triggering the widest range of PTI responses against potential threats. Over the past quarter-century since the first report (Felix et al., 1999), the plant flagellin-sensing mechanisms and signaling pathways have been studied extensively. Furthermore, they have served as a foundation for understanding and expanding our knowledge of plant immune responses to other MAMP signals. In this review, we outline the current understanding of flagellin-sensing mechanisms and immunity in plants with the aim of providing valuable lessons for exploring other PTI responses in plant defense.

## Structural basis of flagellin perception

### Flagellin

Flagellin protein, encoded by *fliC* (or its homologs), serves as the fundamental building block of the bacterial flagellum, a helical filamentous organelle that enables bacteria to propel forward in aqueous environments (Rossez et al., 2015). The primary structure of the flagellin protein is a boomerang-shaped molecule with multiple structural domains, D0–D3 (Samatey et al., 2001). The conserved D0 and D1 inner domains comprise the core channel of the flagellar filament, while the variable D2 and D3 outer domains protrude outward from the core (Yonekura et al., 2003). Both N- and C-terminal chains located in the D0–D1 domain contribute to the formation of α-helical coiled-coil interfaces and hydrophobic interactions, promoting connections with one another for filament polymerization (Yonekura et al., 2003). This pivotal role of the D0–D1 domain in flagellar filament polymerization is highlighted by its evolutionary conservation across flagellated bacterial species (Beatson et al., 2006). Hence, the host’s surveillance system specifically targets these conserved flagellin domains, which are functionally significant for bacterial colonization (Boller and He, 2009).

### Flagellin recognition by host cells

Leucine-rich repeat (LRR) domains have been identified as major docking regions for flagellin in both plant and animal receptors. In animals, the PM-located Toll-like receptor 5 (TLR5) primarily recognizes bacterial flagellin through its extracellular LRR domains (Hayashi et al., 2001). The extracellular LRR domain of TLR5 interacts extensively with the three helices of the flagellin D1 domain, particularly on its lateral side, a critical region for protofilament formation (Smith et al., 2003). The interaction between the flagellin monomer and TLR5 initially occurs in a 1:1 ratio, subsequently leading to the assembly of a secondary dimerization in the form of a 2:2 tail-to-tail signaling complex (Yoon et al., 2012). In addition, an intracellular nucleotide-binding domain leucine-rich repeat (NLR) family member, apoptosis inhibitory protein 5 (NAIP5), binds to both terminal helices of the flagellin D0 domain in the cytosol (Lightfield et al., 2008). This interaction induces a conformational change, leading to the formation of an inflammasome with the recruitment of another NLR family protein, NLRC4 (Tenthorey et al., 2017). Unlike animals, plants rely on PM-located extracellular LRR domain-containing receptor kinases (LRR-RKs) for flagellin sensing (Gómez-Gómez and Boller, 2000). A well-studied example is FLAGELLIN-SENSING2 (FLS2) found in multiple plant species, including *Arabidopsis thaliana* (hereafter *Arabidopsis*) (Rossez et al., 2015). While TLR5 senses flagellin through extensive interaction across the D1 domain, FLS2 recognizes a short peptide spanning a loop between the α-helices of the D0 and D1 domains in the N terminus of flagellin (Yoon et al., 2012; Sun et al., 2013). Many plants recognize a peptide composed of 22 amino acid residues, represented by flg22 (QRLSTGSRINSAKDDAAGLQIA, i.e., from *Pseudomonas aeruginosa*), likely resulting from the degradation of flagellin, through FLS2 orthologs (Table 1) (Meindl et al., 2000; Buscaill et al., 2019). However, the essential peptide length varies among plant species. For instance, tomato FLS2 (SlFLS2) recognizes a smaller part of the flg22 epitope, known as flg15 (RINSAKDDAAGLQIA), which cannot activate *Arabidopsis* FLS2 (Table 1) (Chinchilla et al., 2005; Robatzek et al., 2007; Mueller et al., 2012). In addition, a subset of solanaceous species detect a second epitope of flagellin located in the D1 domain, termed flgII-28, through an independent LRR-RK, FLAGELLIN-SENSING3 (FLS3) (Cai et al., 2011; Hind et al., 2016; Roberts et al., 2020).

**Table 1:** Representative flagellin epitope sequences and their cognate receptors.

Epitope	Amino acid sequence	Representative bacterial species	Receptor	Reference
flg22^Pa^	QRLSTGSRINSAKDDAAGLQIA	*Pseudomonas aeruginosa*	AtFLS2	Gómez-Gómez et al. (1999); Gómez-Gómez and Boller, 2000
flg22^Pto^	TRLSSGLKINSAKDDAAGLQIA	*Pseudomonas syringae* pv. *tomato* DC3000	AtFLS2	Cai et al. (2011)
flg15^Pa^	RINSAKDDAAGLQIA	*P. aeruginosa*	SlFLS2	Felix et al. (1999); Mueller et al. (2012)
flg15^Eco^	RINSAKDDAAGQAIA	*Escherichia coli*	SlFLS2	Meindl et al. (2000); Robatzek et al. (2007)
flgII-28	ESTNILQRMRELAVQSRNDSNSATDREA	*Pseudomonas syringae* pv. *tomato* DC3000	SlFLS3	Cai et al. (2011); Hind et al. (2016)
flg22^Atum^	SRVSSGLRVKSASDNAAYWSIA	*Agrobacterium tumefaciens*	VrFLS2^XL^	Fürst et al. (2020)
flg22^Rso^	QRLSTGLRVNSAQDDSAAYAAS	*Ralstonia solanacearum*	GmFLS2	Meindl et al. (2000); Wei et al. (2020)

Representative flagellin-derived epitope sequences from various bacterial species and their corresponding cognate receptors. For each epitope, the amino acid sequence, sequence length, bacterial source, cognate receptor, and relevant references are provided. Although multiple receptors across diverse plant species can respond to a given epitope, the listed receptors represent those in which recognition was first characterized. At, *Arabidopsis thaliana*; Sl, *Solanum lycopersicum*; Vr, *Vitis riparia*; Gm, *Glycine max*.

FLS2 consists of an extracellular LRR domain (ECD), a single transmembrane domain (TM), a cytoplasmic juxtamembrane domain (cJD), and a cytoplasmic kinase domain (KD) (Table 2) (Ngou et al., 2022). The initial binding of flg22 in *Arabidopsis* occurs on the concave LRR interface of FLS2, spanning from the 3rd to the 16th LRR (Dunning et al., 2007; Sun et al., 2012, 2013). The N-terminal seventeen residues of flg22 have been defined as an “address” segment required to interact with FLS2, while the remaining C-terminal five residues have been defined as a “message” segment, creating a docking site for the co-receptor BRI1-ASSOCIATED KINASE1 (BAK1) (Meindl et al., 2000; Yan et al., 2012; Sun et al., 2013). Most immunogenic flg22 sequences feature the conserved residues Asp^14^ and Asp^15^ in the address segment and Gly^18^ and Leu^19^ in the message segment for interactions with FLS2 and BAK1, respectively (Sun et al., 2013; Colaianni et al., 2021). Flg22-induced FLS2–BAK1 heterodimerization leads to the triggering of a rapid and broad-spectrum immune response (Figure 1). Similarly, TLR5 recognition of flagellin also follows the address/message paradigm: the D1 domain functions as the address for initial pattern recognition, while regions within the D0 domain serve as the message required for receptor dimerization and full signaling activation. This concept helps to explain why the flagellin of the human pathogen *Salmonella enterica*, which contains intact D1 and D0 domains, efficiently activates TLR5 (Yoon et al., 2012). By contrast, *Helicobacter pylori* flagellin evades TLR5 detection through mutations in the D1 address region (Andersen-Nissen et al., 2005; Kreutzberger et al., 2020), and flagellins from human gut commensal bacteria adopt a different strategy: they retain a functional D1 address that allows TLR5 binding, but they lack the appropriate D0 message for dimerization, leading to significantly attenuated signaling responses (Clasen et al., 2023). Strategic alterations in the “address” and “message” segments of flg22 to evade FLS2 detection during plant host–bacteria interactions will be discussed further in the section titled Arms races in flagellin-sensing mechanisms.

**Table 2:** Critical functional residues and modifications of FLS2

Residue	Region	PTM	Responsible factor	Method	Molecular function	Reference
N179	ECD(LRR4)	(putative) N-glycosylation	?	SDM	flg22-binding, FLS2–FLS2 association	Sun et al. (2012)
R294	ECD(LRR9)	–	flg22	structure-based SDM (FLS2–flg22–BAK1 complex)	flg22-binding, FLS2–BAK1 interaction	Sun et al. (2013); Lee et al. (2024)
H316	ECD(LRR10)	–	flg22	structure-based SDM (FLS2–flg22–BAK1 complex)	flg22-binding, FLS2–BAK1 interaction	Sun et al. (2013)
G318	ECD(LRR10)	–	flg22	EMS mutagenesis (fls2-24)	flg22-binding, signaling	Gómez-Gómez and Boller, 2000
T342	ECD(LRR11)	–	flg22	structure-based SDM (FLS2–flg22–BAK1 complex)	flg22-binding, FLS2–BAK1 interaction	Sun et al. (2013)
T366	ECD(LRR12)	–	flg22	Ala-scanning mutagenesis	flg22-binding, signaling	Dunning et al. (2007)
N388	ECD(LRR13)	(putative) N-glycosylation	?	SDM	flg22-binding, FLS2–FLS2 association	Sun et al. (2012)
S390	ECD(LRR13)	–	flg22	Ala-scanning mutagenesis	flg22-binding, signaling	Dunning et al. (2007)
H392	ECD(LRR13)	–	flg22	Ala-scanning mutagenesis	flg22-binding, signaling	Dunning et al. (2007)
D414	ECD(LRR14)	–	flg22	Ala-scanning mutagenesis	flg22-binding, signaling	Dunning et al. (2007); Sun et al. (2013)
S437	ECD(LRR15)	–	flg22	SDM	flg22-binding, signaling	Sun et al. (2013)
G493	ECD(LRR17)	–	flg22	FLS2 natural variation	flg22-binding, signaling	Vetter et al. (2012)
C830	JXD	S-acylation	(putative) BAK1, PUB12/13	SDM	signaling	Hemsley et al. (2013); Hurst et al., 2019
C831	JXD	S-acylation	(putative) BAK1, PUB12/13	SDM	signaling	Hemsley et al. (2013); Hurst et al., 2019
T867	JXD	(putative) phosphorylation	?	SDM	signaling, FLS2 endocytosis, FLS2–FLS2 association	Robatzek et al. (2006); Sun et al. (2012)
S869	JXD	phosphorylation	BAK1	MS	?	Xu et al. (2013)
S878	KD	(putative) phosphorylation	?	SDM	signaling	Robatzek et al. (2006)
S906	KD	phosphorylation	BAK1, BIK1	MS	?	Yan et al. (2012); Xu et al. (2013)
S909	KD	phosphorylation	?	MS (*in vitro*), SDM	signaling	Cao et al. (2013)
S938	KD	phosphorylation	BIK1	MS (*in vitro*), SDM	BIK1 phosphorylation, signaling	Cao et al. (2013); Xu et al. (2013)
T941	KD	phosphorylation	BIK1	MS	?	Xu et al. (2013)
S961	KD	phosphorylation	BAK1	MS	?	Yan et al. (2012)
D997	KD	phosphorylation	BIK1	SDM	BIK1 phosphorylation	Cao et al. (2013)
S1014	KD	phosphorylation	BAK1	MS	?	Yan et al. (2012)
T1040	KD	phosphorylation	?	SDM	signaling	Robatzek et al. (2006)
G1064	KD	phosphorylation	?	EMS mutagenesis (fls2-0 and fls2-17)	FLS2–FLS2 association, signaling	Gómez-Gómez and Boller, 2000; Gómez-Gómez et al., 2001; Sun et al. (2012); Robatzek and Wirthmueller, 2013
T1072	KD	phosphorylation	?	SDM	signaling	Robatzek et al. (2006)
S1084	KD	phosphorylation	BIK1	MS (*in vitro*), SDM	signaling	Cao et al. (2013); Xu et al. (2013)
S1115	KD	phosphorylation	BAK1, BIK1	MS	?	Yan et al. (2012); Xu et al. (2013)
K1120	KD	SUMOylation	BIK1	SDM	BIK1 release	Orosa et al. (2018)
C1132	KD	S-acylation	BAK1, PUB12/13	SDM	FLS2–BAK1 interaction, FLS2 maintenance	Hurst et al. (2023)
C1135	KD	S-acylation	BAK1, PUB12/13	SDM	FLS2–BAK1 interaction, FLS2 maintenance	Hurst et al. (2023)

The functional residues of FLS2 were identified through various experimental approaches. Each residue is annotated with its location, type of post-translational modification (PTM), interacting factors, experimental method used for identification, and observed molecular function. ECD, extracellular domain (97–769 aa); TM, transmembrane domain (802–827 aa); JXD, cytosolic juxtamembrane domain (828–869 aa); KD, kinase domain (870–1173 aa); SDM, site-directed mutagenesis; MS, mass spectrometry; LRR, leucine-rich repeat.

**Figure 2:**
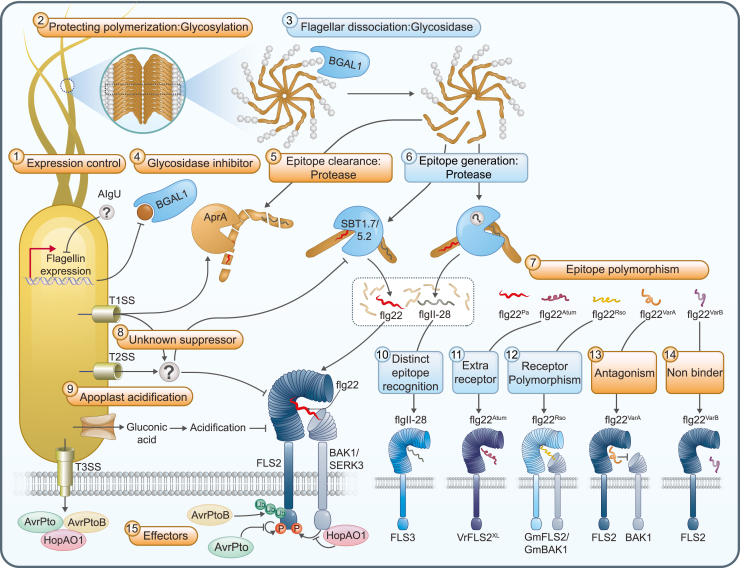
Processing and sensing of the flagellin epitope: The tit-for-tat arms race between plants and bacteria. The brown box indicates bacterial strategies to evade the plant immune system, and the blue box indicates the plant’s strategies to detect flagellin epitopes. (1) When bacteria arrive, flagellin expression is downregulated to reduce the exposure of flagellin epitopes. (2) Surface flagellin molecules are glycosylated to prevent filament dissociation. (3) Plants counteract flagellin glycosylation by secreting glycosidases such as BGAL1. (4) Some bacterial strains counteract BGAL1 by secreting small inhibitory molecules, which are characterized as heat-stable, small hydrophilic compounds that specifically prevent BGAL1 activity through as-yet-uncharacterized mechanisms. (5) To clear flagellin epitopes, dissociated flagellin monomers are degraded by bacterial proteases secreted through the type I secretion system (T1SS). (6) By contrast, dissociated flagellin monomers are processed into peptide epitopes like flg22 by apoplastic plant proteases such as SBT1.7/5.2, whereas flgII-28 is processed by unknown proteases. (7) Epitope sequence polymorphism can impair interaction with flagellin receptors. (8) Specific pathovars secrete unidentified suppressor molecules through the T1SS or T2SS to suppress flg22-induced activation of FLS2. (9) Apoplast acidification by bacterially secreted gluconic acid also suppresses flg22-induced activation of FLS2. (10) Some plant species employ additional flagellin receptors that recognize a distinct epitope, flgII-28. (11) *Vitis riparia* uses an additional FLS2 receptor to detect flg22^Atum^ sequences that differ significantly from the flg22^Pa^ sequence. (12) *Glycine max* exhibits receptor polymorphism that enables it to recognize a polymorphic flg22^Rso^ sequence. (13) Some sequence polymorphisms in flg22 can interact with FLS2 but lead to the blocking of BAK1 recruitment. (14) Some sequence polymorphisms in flg22 can prevent binding with FLS2. (15) Bacteria inject effector molecules into the cytosol through the T3SS to suppress flg22-induced activation of FLS2.

Despite extensive research on FLS2 signaling using flg22 peptides, the identity of the protease responsible for the proteolytic release of flg22 epitopes from flagellins had remained a long-standing question (Buscaill et al., 2019). Recently, Matsui et al. identified two *Arabidopsis* subtilases, SBT5.2 and SBT1.7, that exhibit specific proteolytic activity at the C terminus of the flg22 epitope in flagellin (Figure 2). Given the significance of the message segment for BAK1 recruitment, proper C-terminal cleavage of the flg22 epitope is required for signaling, rather than N-terminal cleavage. In particular, the well-conserved P2 position Ile^21^ in the message segment is a pivotal residue for efficient cleavage by SBT5.2, enabling cleavage of flagellin derived from various bacterial species (Matsui et al., 2024). In support of these findings, Buscaill et al. (2024) further demonstrated that, in *Nicotiana benthamiana*, NbSBT5.2 not only contributes to the processing of flg22 epitopes but also facilitates the rapid inactivation of flg22 by cleaving within the flg22 epitope, particularly after Asn^10^, thereby enabling spatiotemporal regulation of immune responses. Most recently, a proteomic analysis of proteins from plant apoplastic fluid revealed that extracellular proteasomes participate in the proteolytic processing of flg22 epitopes from bacterial flagellin, together with other proteases (Karimi et al., 2025).

## FLS2-dependent PTI events

During pathogen invasion attempts, numerous molecular patterns are released from exogenous (non-self) and/or endogenous (self) sources (Boutrot and Zipfel, 2017; Tanaka and Heil, 2021). These molecular patterns are recognized by the extracellular domains of one or more PRRs in plants, driving PTI responses. PTI responses encompass a broad range of signaling outputs that are sufficient to deter nonspecialized attackers (Lee et al., 2021). Because FLS2 is the most extensively studied plant PRR to date, the most general events in PTI responses have been established from FLS2 studies. PTI includes a series of sequential and multiple simultaneous events. Early PTI events include non-generic responses such as activation of receptor-like cytoplasmic kinases (RLCKs), activation of ion channels, accumulation of reactive oxygen intermediates (ROIs), and activation of defense-specific mitogen-activated protein kinases (MAPKs). The collective output of early PTI responses results in extensive transcriptional regulation to orchestrate a delicate balance between growth- and defense-related gene expression, thereby empowering the plant to withstand pathogen attacks (Figure 1).

### RLCK activation

When flg22 binds to the extracellular LRR region of FLS2, the C-terminal residues of flg22 create a docking site for BAK1, facilitating formation of a heterodimeric FLS2–BAK1 immunocomplex (Sun et al., 2013). This interaction between the LRR domains of the two receptors brings their cytoplasmic kinase domains into proximity, initiating a signaling cascade through auto- and transphosphorylation events (Cao et al., 2013; Sun et al., 2013; Ngou et al., 2022). The activated receptor complex subsequently phosphorylates RLCKs, especially those from the RLCK-VII and RLCK-XII families, which play pivotal roles in downstream signaling events. Among the RLCK families, *Botrytis*-induced kinase 1 (BIK1) and PBS1-like kinase (PBL1) act as key components downstream of the flg22-induced immune response (Wan et al., 2019). BIK1 associates with both FLS2 and BAK1 in their unstimulated states (Lu et al., 2010; Zhang et al., 2010). FLS2 kinase activity is required for FLS2–BIK1 association in the resting state but not for heterodimeric immunocomplex formation between FLS2 and BAK1 upon flg22 (Cao et al., 2013). After heterodimeric complex formation, BIK1 is rapidly phosphorylated at the activation-loop Ser236 and Thr237 residues by BAK1 (Table 2) (Lu et al., 2010; Feng et al., 2012). Although FLS2 does not appear to function as a direct protein kinase for BIK1, phosphorylation of BIK1 is dependent on the protein kinase activity of FLS2, relying in particular on the Ser938 autophosphorylation site of FLS2 (Table 2) (Cao et al., 2013). In addition, activated BIK1 transphosphorylates both FLS2 and BAK1 to fully activate them, and BIK1 then dissociates from the FLS2–BAK1 complex to initiate ROI production. The dissociation of BIK1 from the FLS2–BAK1 complex is regulated by monoubiquitination of BIK1 through the *Arabidopsis* E3 ubiquitin ligases RING-H2 FINGER A3A (RHA3A) and RHA3B (Ma et al., 2020). SUMOylation of FLS2-KD is also involved in the dissociation of BIK1 from the FLS2–BAK1 complex, which is reversed by the deSUMOylation protease Desi3a (Orosa et al., 2018). A recent study demonstrated that flg22 treatment leads to rapid accumulation of nitric oxide (NO) within 10 min of perception, although the precise molecular mechanisms of NO synthesis still remain to be identified. This NO accumulation subsequently results in S-nitrosylation of BIK1 at Cys^80^, which is required for its activation and stabilization, thereby enhancing ROI production (Cui et al., 2024).

The precise control of BIK1 protein levels is maintained through a complex interplay of various regulatory mechanisms for immune-system homeostasis (DeFalco and Zipfel, 2021). In the resting state, BIK1 activation is prevented by dephosphorylation via the PP2C-type protein phosphatase PP2C38 (Couto et al., 2016). Non-activated BIK1 is polyubiquitinated by the E3 ubiquitin ligases PLANT U-BOX PROTEIN 25 and 26 (PUB25/26) for proteasomal degradation (Wang et al., 2018). The activity of PUB25/26 is promoted through phosphorylation by calcium-dependent protein kinase 28 (CPK28), which attenuates BIK1-mediated immune signaling (Monaghan et al., 2014; Wang et al., 2018). Recent research has shown that polyubiquitination of non-activated BIK1 is mediated not only by PUB25/26 but also by another E3 ubiquitin ligase, PUB4 (Yu et al., 2022). Regulation of BIK1 stabilization is important for effective immune signaling. PUB4 promotes the degradation of non-activated BIK1, and activated BIK1 stabilization is further maintained by phosphorylation events mediated by MAPKKKKs (MAP4Ks), specifically MAP4K3/SIK1 and MAP4K4 (Zhang et al., 2018; Jiang et al., 2019). Upon perception of flg22 by FLS2, MAP4K4 phosphorylates PP2C38, resulting in the dissociation of PP2C38 from BIK1 to prevent dephosphorylation (Jiang et al., 2019).

### Cytosolic Ca^2+^ influx

The movement of calcium ions (Ca^2+^) is a universal signaling event in cell biology. Ca^2+^ acts as a secondary messenger that triggers a cascade of intracellular processes. During PTI events, a prompt and substantial increase in cytosolic Ca^2+^ concentration occurs in plant cells. The flg22-induced Ca^2+^ influx begins at ∼30 s to 2 min and reaches its peak at around 4–6 min after flg22 treatment (Bigeard et al., 2015). Multiple Ca^2+^ channels are involved in the overall pattern-induced calcium response. Cyclic nucleotide-gated channel 2 and 4 (CNGC2 and CNGC4) form a functional channel when combined, which is blocked by calmodulin (CaM) in the resting state. During the PTI response, the CaM-gated CNGC2–CNGC4 channel is phosphorylated by BIK1, leading to Ca^2+^ entry into the cytosol (Tian et al., 2019). The glutamate receptor 2 (GLR2) clade members GLR2.7, 2.8, and 2.9 are Ca^2+^-permeable channels that contribute to pattern-induced Ca^2+^ influx (Bjornson et al., 2021). The increased Ca^2+^ is monitored by cytosolic Ca^2+^ sensors, calcium-dependent protein kinases (CPKs). CPK4, CPK5, CPK6, and CPK11 mediate various downstream immune responses (Boudsocq et al., 2010; Dubiella et al., 2013; Kadota et al., 2014). A recent study demonstrated that activated BIK1/PBL1 phosphorylates and thereby activates vacuolar Ca^2+^/H^+^ exchangers to process excessive cytosolic Ca^2+^ (Wang et al., 2024b).

### Reactive oxygen intermediates (ROIs) accumulation

The robust accumulation of extracellular ROIs is a widely observed early signaling response during immune processes in both mammals and plants (Torres and Dangl, 2005). ROI production begins within 2–3 min after flg22 treatment, peaks between 10 and 20 min, and ends within 40–50 min in luminol-based assays (Lozano-Durán and Belkhadir, 2017). The resulting ROIs function as potent weapons in the plant’s defense repertoire. ROIs have multiple roles in the immune response, acting as cytotoxic agents and strengthening the plant cell wall to block invading pathogens (Torres and Dangl, 2005). PM-located NADPH oxidases, particularly respiratory burst oxidase homolog (RBOH) proteins, are responsible for producing superoxide anions (O_2_^−^) in biotic interactions, which can be converted to hydrogen peroxide (H_2_O_2_) by peroxidases (Torres and Dangl, 2005). Among the 10 RBOH proteins in *Arabidopsis*, RBOHD has a predominant function in generating the majority of ROIs in PTIs, whereas RBOHF is primarily involved in regulating the hypersensitive response (Torres and Dangl, 2005; Nühse et al., 2007; Zhang et al., 2007). RBOHD consists of a long N-terminal extension with two EF hands that bind cytosolic Ca^2+^, six transmembrane domains, and a C-terminal extension with NADPH and FAD binding domains (Torres and Dangl, 2005). Phosphoproteomic analyses after flg22 treatment have demonstrated that RBOHD undergoes extensive phosphorylation at the long cytosolic N-terminal extension, which is necessary for RBOHD activation (Benschop et al., 2007; Nühse et al., 2007). Upon flg22 perception, activated BIK1 directly phosphorylates the N-terminal Ser^39^, Ser^343^, and Ser^347^ residues of RBOHD, leading to induction of ROI production (Kadota et al., 2014; Li et al., 2014). In addition, MAP4K4/SIK1 directly associates with RBOHD and phosphorylates the N-terminal Ser^8^, Ser^9^, Ser^339^, and Ser^347^ residues to enhance ROI production (Zhang et al., 2018). The N-terminal Ser^133^/Ser^163^(Thr^161^/Ser^162^)/Ser^347^ residues of RBOHD are also phosphorylated by activated CPK4, CPK5, CPK6, and CPK11 in response to elevated cytosolic Ca^2+^ (Boudsocq et al., 2010; Dubiella et al., 2013; Kadota et al., 2014). Because Ser^347^ can be phosphorylated by BIK1, MAP4K4/SIK1, and CPKs, it holds particular significance in activating RBOHD to promote ROI production in the PTI response. In addition to N-terminal regulation, the protein stability of RBOHD can be controlled through its C-terminal extension. The C-terminal extension of RBOHD is phosphorylated and polyubiquitinated in the resting state by PBL13 and PBL13-interacting RING domain E3 ligase (PIRE), respectively. This process leads to endocytosis and vacuolar degradation of RBOHD. Upon flg22-induced immune responses, PIRE is dynamically phosphorylated by an unknown kinase, which inhibits PIRE-mediated RBOHD endocytosis. This regulation prevents RBOHD degradation, enabling fine-tuned production of ROIs during PTI responses (Lee et al., 2020).

### MAPK activation

MAPK cascades are evolutionarily conserved modules in eukaryotic cells that transmit and amplify signals from upstream receptors to downstream targets (i.e., transcription factors), thereby regulating cellular responses (Ichimura et al., 2002). A series of phosphorylation events, involving MAPK kinase kinases (MAPKKKs/MEKKs), MAPK kinases (MAPKKs/MKKs/MEKs), and MAPKs/MPKs, occurs in response to various biotic/abiotic stresses and growth/developmental processes (Sun and Zhang, 2022). Upon flg22 perception, activation of the MAPKKK3/5–MKK4/5–MPK3/6 cascade and the MEKK1–MKK1/2–MPK4 cascade occurs within 5–30 min to induce immune responses (Asai et al., 2002; Qiu et al., 2008; Bi et al., 2018).

RLCKs are recognized as upstream regulators of MAPK activation in PTI signaling. For example, sextuple mutants of members of the RLCK-VII-4 subgroup (*PBL19*, *PBL20PBL37PBL38PCRK1*, and *PCRK2*) exhibit a strong reduction in chitin-induced MAPK activation (Rao et al., 2018). However, double (*BIK1* and *PBL1*), triple (*BIK1*, *PBL1*, and *PBL11*), and quadruple mutants (*BIK1*, *PBL1*, *PBL9*, and *PBL11*) of the RLCK-VII-8 subgroup display normal flg22-induced MAPK activation (Feng et al., 2012; Rao et al., 2018). These results suggest possible higher-order redundancies between RLCK subgroups and/or other regulatory mechanisms in flg22-induced MAPK activation. In addition, a recent study revealed that BRI1-SUPPRESSOR1 (BSU1) family phosphatases participate in flgaellin signaling. BSU1 is phosphorylated by BIK1 at Ser251 to mediate MAPK cascades and immune responses through pathway-specific phosphocodes distinct from brassinosteroid signaling (Park et al., 2022).

### G protein

G proteins play important roles in a wide range of biological signaling pathways in eukaryotic organisms. Whereas activation of G proteins in animals and fungi relies on the ligand-induced guanine nucleotide exchange activity of seven-transmembrane G-protein-coupled receptors, plant G proteins are recognized to be self-activating. In the pre-activation state, GDP-bound Gα interacts with the Gβγ dimer, forming a heterotrimeric complex. Upon activation, Gα exchanges GDP for GTP, leading to its dissociation from Gβγ. Both dissociated Gα and Gβγ can then regulate downstream effector proteins to propagate intracellular signaling. The intrinsic GTPase activity of Gα hydrolyzes GTP to GDP, resulting in the reassociation of Gα with Gβγ. The *Arabidopsis* genome encodes a canonical Gα subunit (GPA1), a Gβ subunit (AGB1), three Gγ subunits (AGG1, AGG2, and AGG3), and three non-canonical extra-large Gα subunits (XLG1, XLG2, and XLG3). Heterotrimeric G proteins with the non-canonical Gα subunits (XLG1/2/3–AGB1–AGG1/2) are required for flg22-induced ROI accumulation and pathogen resistance but not for flg22-induced MAPK activation (Liu et al., 2013; Maruta et al., 2015). Furthermore, these heterotrimeric G proteins stabilize BIK1 through direct inhibition of PUB25/26 in the resting state (Wang et al., 2018). RGS1, a GTPase-accelerating protein (GAP), functions as a negative regulator of the Gα subunit and maintains inactive G proteins through its GAP activity in complex with FLS2 (Liang et al., 2018). Upon flg22 perception, FLS2 triggers BIK1-mediated phosphorylation of RGS1, resulting in RGS1 dissociation from FLS2, and G proteins are activated (Liang et al., 2018).

### Transcriptional reprogramming

The signal transduction triggered by flagellin perception eventually results in extensive transcriptional reprogramming in plant cells, with early changes sharing common features of the plant’s general stress response. As an early step in regulation, transcription factors (TFs) from the CaM-binding transcriptional activator (CAMTA) family are activated in response to elevated cytosolic Ca^2+^ levels (Benn et al., 2014; Bjornson et al., 2021). These CAMTA-family TFs bind to the consensus *cis*-element (vCGCGb) found in genes that are rapidly and transiently induced within 5–10 min after flg22 treatment (Benn et al., 2014; Bjornson et al., 2021). Starting at around 10–30 min after flg22 treatment, genes involved in signaling and transcriptional regulation are significantly upregulated. Notably, these genes often feature binding sites (W-box; TTGACT/C) for WRKY TFs within their regulatory region (Birkenbihl et al., 2017). Moreover, the presence of several W-box motifs within the promoters of WRKY family TFs suggests that WRKY TFs engage in self-regulation, forming part of a feedback mechanism (Birkenbihl et al., 2018).

WRKY TFs are regulated through phosphorylation by multiple kinases, creating a complex regulatory network. Several WRKY TFs, including WRKY33 and WRKY46, have been identified as direct substrates of MPK3/6 (Mao et al., 2011; Sheikh et al., 2016). The phosphorylation of WRKY TFs can lead to diverse functional effects. For instance, when MPK3 phosphorylates WRKY46, this modification leads to the destabilization of WRKY46 upon PMAP perception (Sheikh et al., 2016). Moreover, MPK3/6 phosphorylation enhances the transactivation activity of WRKY33, and CPK5/6 phosphorylation at different sites increases its DNA-binding activity (Zhou et al., 2020b). These diverse phosphorylation events enable specific WRKY modules to be selectively activated and regulate distinct sets of defense genes. MPK3/6-activated WRKYs primarily upregulate defense-related genes, whereas MPK4 signaling can suppress or modulate the expression of other gene subsets (Sheikh et al., 2016). The combinatorial effects of transcriptional feedback regulation—through W-box motifs present in WRKY promoters—and multilayered post-translational modifications enable plants to generate stimulus-specific transcriptional responses. However, functional redundancy among WRKY family members continues to present challenges for characterizing the specific contributions of individual WRKYs to these complex regulatory networks.

### Secondary metabolites and defense hormones

Flg22-induced transcriptional reprogramming accompanies the upregulation of genes involved in secondary metabolite biosynthesis and stress-hormone-related processes. Plants synthesize structurally diverse defense metabolites *de novo*, known as phytoalexins, when challenged by microorganisms (Piasecka et al., 2015). Most phytoalexins isolated from Brassicaceae species are indolic compounds derived from tryptophan metabolism, with camalexin, the major phytoalexin in *Arabidopsis*, accumulating strongly during defense responses (Klein and Sattely, 2017; Zhou et al., 2020b). Many cytochrome P450 enzyme genes are involved in camalexin biosynthesis, such as *CYP71A12*, *CYP71A13*, and *CYP71B15* (*PAD3*), which are highly upregulated upon flg22 treatment (Birkenbihl et al., 2017). WRKY TFs play key roles in regulating these genes and upregulate MYB TFs, particularly MYB51, for flg22 signaling, affecting the biosynthesis of indole glucosinolates as well (Frerigmann et al., 2016; Birkenbihl et al., 2017).

Stress-hormone-related processes are also regulated by WRKY TFs and other TFs for long-term and systemic defense responses (Pandey and Somssich, 2009). Genes involved in hormone biosynthesis and signaling components are upregulated during PTI events, including those responsible for major defense hormones such as ethylene, salicylic acid (SA), and jasmonic acid (JA) (Birkenbihl et al., 2017). Furthermore, the rate-limiting enzymes of ethylene biosynthesis, 1-aminocyclopropane-1-carboxylic acid synthases (ACSs), are directly regulated by MPK6 through phosphorylation-mediated stabilization, indicating early activation of ethylene signaling during PTI responses (Liu and Zhang, 2004; Joo et al., 2008). As ethylene signaling modulates JA and SA signaling pathways, its early activation enables it to serve as a coordinator of subsequent defense responses, integrating other signaling pathways (Broekgaarden et al., 2015).

### Callose deposition

Callose deposition is induced within 8–12 h of flg22 treatment and is dependent on the PM-located callose synthase Powdery Mildew Resistance 4 (PMR4) (Jacobs et al., 2003; Clay et al., 2009). Callose is a β-(1,3)-D-glucan polysaccharide with β-1,6-branches, known for its functions as a physical stiffener and/or sealant in defense mechanisms. However, the exact effect of callose on structural properties of the plant cell wall remains unclear (Wang et al., 2021). Although flg22-induced callose deposition depends on MYB51/ethylene-induced indole-3-glucosinolate pathways, the mechanism by which these upregulated indole glucosinolates affect callose biosynthesis remains to be clarified (Clay et al., 2009). Notably, flg22-induced callose deposition was observed in *cyp71A12*, *cyp71A13*, and *pad3* mutants, indicating that camalexin biosynthesis is not required for callose deposition (Clay et al., 2009).

### Growth inhibition

**Figure 3:**
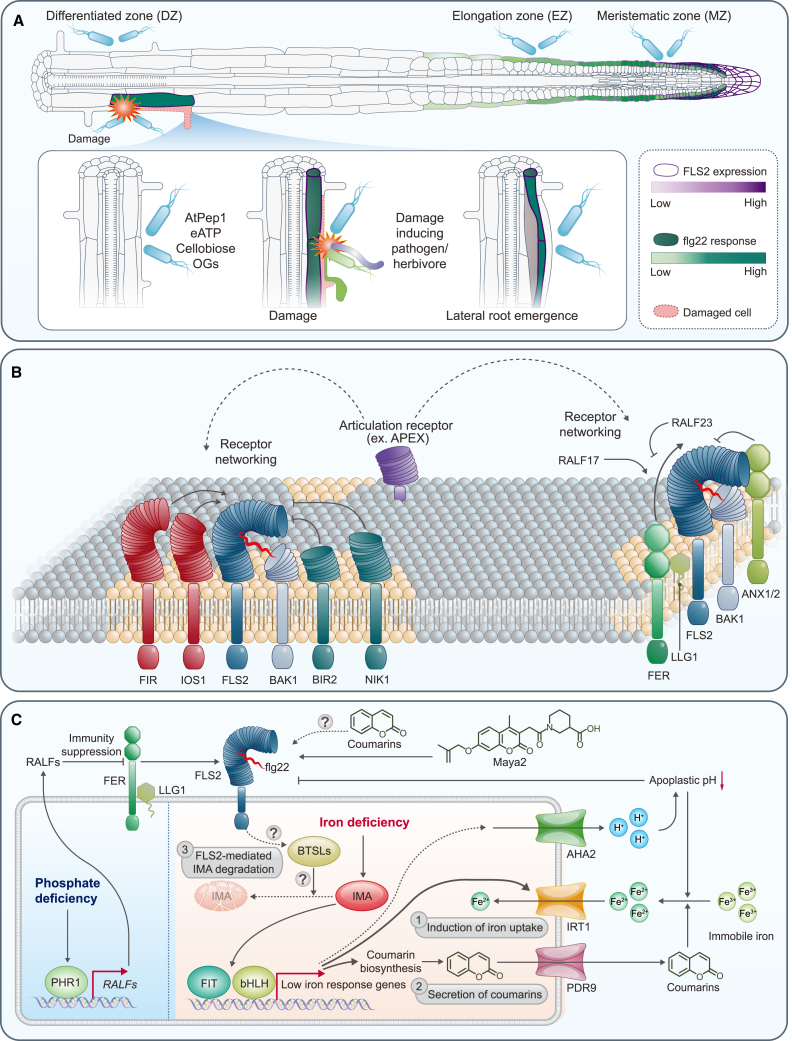
Emerging topics in flagellin signaling. **(A)** Representative tissue-specific FLS2 activation in the *Arabidopsis* root. FLS2 expression and flg22 response levels are shown as purple and green color gradients, respectively. Flg22 responses are observed in epidermal cells of the meristematic and elongation zones, but they are not observed in the differentiated zone (DZ). However, when outer cells are damaged, inner adjacent cells induce FLS2 expression and can thereby respond to flg22. Known damage-associated molecular pattern molecules such as AtPep1, extracellular ATP (eATP), cellobiose, and oligogalacturonides (OGs) do not induce this response; only physical damage can trigger it. A similar phenomenon is observed during lateral root emergence. **(B)** Receptor networking and membrane nanodomains. Putative membrane nanodomains are indicated in yellow. Representative *Arabidopsis* RKs that modulate the function of flg22-induced FLS2–BAK1 complex formation are displayed. The LRR-RKs BIR2 and NIK1 inhibit complex formation, whereas FIR and IOS1 stimulate complex formation. The CrRLK1L receptor FER and its co-receptor LLG1 act as positive regulators for complex formation, whereas ANX1/2 act as negative regulators. The positive regulatory role of the FER–LLG1 in complex formation is inhibited by the perception of RALF23 and stimulated by the perception of RALF17. **(C)** Representative effects of environmental conditions on the flagellin-sensing mechanism. During phosphate deficiency (left), the transcription factor (TF) PHR1 induces inhibitory RALF peptides, which suppress FLS2 activity through interaction with FER–LLG1. During iron deficiency (right), the IMA signaling pathway activates FER-like iron deficiency-induced TF (FIT) and bHLH TFs, leading to the induction of the *Arabidopis* H^+^-ATPase isoform 2 (AHA2), the Fe^2+^ transporter IRT1, and the coumarin biosynthesis pathway. Coumarin molecules are transported to the apoplast via PDR9, increasing the solubility of immobile iron. FLS2 signaling is involved in the degradation of IMA through the activation of BTSLs. A weak FLS2 agonist, Maya2, shares the core structure of coumarins, suggesting a possible relationship between coumarins and FLS2 activity.

Plant defense mechanisms often entail growth alterations upon recognition of MAMPs. Such growth inhibition largely depends on the expression patterns of PRRs. Since *FLS2* is expressed ubiquitously across all *Arabidopsis* tissues, growth inhibition can be observed in both leaves and roots (Beck et al., 2014). Although both the phyllosphere and rhizosphere host a diverse array of microorganisms (Bai et al., 2015), the interaction between plant roots and rhizosphere microorganisms is particularly dynamic (Wippel et al., 2021), leading to fine-tuned, cell-type-specific immune responses to optimize resource allocation and avoid constitutive responses to commensal rhizosphere microorganisms. Growth inhibition tends to be less pronounced in roots than in leaves (Gómez-Gómez et al., 1999), largely owing to the lower expression of *FLS2* in roots. *FLS2* expression exhibits a tissue-restricted pattern; it is found primarily in the differentiated stele but is also weakly expressed in all root tissues (Rich-Griffin et al., 2020; Emonet et al., 2021). The induction patterns of downstream defense marker genes—confined to the root cap and transition/elongation zone—largely correspond to the expression pattern of *FLS2*, although there are some discrepancies. Specifically, despite high expression of *FLS2* in the differentiated stele, marker genes are not induced because endodermal barriers such as the Casparian strip and suberization block the entry of flg22 (Emonet et al., 2021). Although differentiated roots lack responses to flg22 treatment, sporadic cell death resulting from emerging lateral roots or local wounding induces flg22 responses in neighboring cells, accompanied by localized *FLS2* induction (Figure 3A) (Zhou et al., 2020a). Furthermore, ectopic expression of *FLS2* in the meristematic epidermis results in flg22 hypersensitivity and meristem collapse, indicating that precise spatial regulation of *FLS2* expression is essential to prevent severe root growth inhibition in response to rhizosphere bacteria.

### Stomatal closure

Stomatal closure is a critical defense mechanism because open stomata can serve as entry points for pathogen invasion (Melotto et al., 2006). Upon flg22 perception in guard cells, cytosolic Ca^2+^ influx occurs within 5 min via the Ca^2+^ channel OSCA1.3 (Thor et al., 2020). The elevated cytosolic Ca^2+^ concentration activates CPKs and calcineurin B-like interacting protein kinases, leading to the opening of S-type anion channels such as slow anion channel 1 (SLAC1) and SLAC1 homolog 3 (SLAH3) (Deger et al., 2015). Consequently, a transient efflux of anions results in membrane depolarization, ultimately causing stomatal closure through a decrease in guard-cell turgor pressure. In response to flg22 treatment, genes encoding small phytocytokine peptides called small phytocytokines regulating defense and water loss (SCREWs) are upregulated. The SCREWs regulate stomatal re-opening through receptor-mediated signaling (Liu et al., 2022), a topic that will be discussed in detail in the section titled The emerging field of flagellin signaling.

### Other responses

In addition to the well-defined categories of PTI responses, recent studies have highlighted additional FLS2-mediated immune responses. Phytopathogens intercept photosynthetic sugars during apoplastic transport in the plant host, using them as carbon sources for their growth and virulence. To counteract this interception, flg22 enhances apoplastic sugar retrieval through BAK1-mediated phosphorylation of the PM-localized sugar transporter protein STP13 (Yamada et al., 2016). Recently, a follow-up study revealed that this enhanced sugar uptake leads to elevated cellular levels of glucose-6-phosphate, which coordinates plant antibacterial defense responses by promoting SA synthesis pathways (Yamada and Mine, 2024). In addition, a recent study suggested that various MAMPs induce a transient spike in phosphatidic acid (PA) regulated by dual phosphorylation of diacylglycerol kinase 5 (DGK5). This DGK5-mediated PA production is regulated in opposite ways: positively by BIK1 and negatively by MPK4. The accumulated PA is crucial for robust PTI responses, as it binds to the N-terminal quadruple arginine residues of RBOHD (Arg^149^, Arg^150^, Arg^156^, and Arg^157^), stabilizing RBOHD from ubiquitination-mediated protein degradation (Kong et al., 2024).

### Signal termination

The activation of flagellin receptors needs to be properly modulated to prevent excessive immune responses and maintain cellular homeostasis. Given the importance of RK phosphorylation in activating downstream signaling, one modulation mechanism involves protein phosphatase-mediated dephosphorylation of the flagellin receptor complex. For instance, the Ser/Thr phosphatase PP2A, comprising the subunits A1, C4, and B′η/ζ, regulates BAK1 phosphorylation (Segonzac et al., 2014). In addition, PP2C38 negatively regulates the flg22-induced immune response by dephosphorylating BIK1 (Couto et al., 2016). Another mechanism includes modulation of the receptor complex by endocytosis. *Arabidopsis* FLS2 is constitutively recycled by endocytosis in a Brefeldin A (BFA)-sensitive manner in the absence of flg22. However, in the presence of flg22, FLS2 undergoes ligand-dependent endocytosis in a BFA-insensitive manner, which requires Rab5 GTPase activity (Beck et al., 2012). A mutation at Thr867 in the cJD of FLS2 impairs ligand-dependent endocytosis, and Ser878 has been identified as a BIK1 phosphorylation site by phosphoproteomic analysis, suggesting that phosphorylation of the cJD of FLS2 by BIK1 may be required for ligand-dependent endocytosis of FLS2 (Robatzek et al., 2006; Xu et al., 2013). Furthermore, post-translational modifications participate in the negative regulation of FLS2 by regulating its stability. Ubiquitin plant U-box E3 ligases (PUB12 and 13) are activated by BAK1-mediated phosphorylation and polyubiquitinate FLS2, thereby leading to FLS2 degradation (Lu et al., 2011). Recently, ligand-dependent S-acylation of FLS2 at cysteine residues in the KD (Cys1132, 1135) was shown to occur in a PUB12/13- and BAK1-dependent manner, suggesting that S-acylation of FLS2 contributes to its stability (Hemsley et al., 2013; Hurst et al., 2023).

## Arms races in flagellin-sensing mechanisms

Although flagellar-based motility provides an advantage for successful colonization within the host for several bacteria, this crucial function becomes a liability when facing the activities of the host immune system. Bacteria employ various evasion tactics to overcome the host immune system, including concealment, camouflage, and deception against flagellin sensors. In response, plants have engaged in an evolutionary arms race, developing tit-for-tat strategies to counter these bacterial tactics and enhance their own immune defenses (Figure 2).

### Concealment

Once bacteria have reached their intended site and motility becomes unnecessary, they downregulate flagellar expression to conceal the exposure of MAMP epitopes (Sanguankiattichai et al., 2022). Transcriptomic analysis of *Pseudomonas syringae* pv. *syringae* B728a cells in different plant habitats revealed reduced expression of flagellar motility-related genes when the bacteria successfully entered the leaf apoplast (Yu et al., 2013). Although the extracytoplasmic sigma factor AlgU is proposed to be required for downregulation of flagellin gene expression (Bao et al., 2020), the precise environmental cues and other signaling factors responsible remain unclear. Given the overexpression of flagellar genes observed in the gut microbiota of TLR5^−/−^ mice compared with wild-type mice, the PTI response may be involved in this regulation (Cullender et al., 2013). Indeed, the plant’s PTI response results in substantial transcriptomic changes in *P. syringae* pv*. tomato* DC3000 (*Pto*), including alterations in flagellar biosynthesis genes (Nobori et al., 2018). However, given the exquisite sensitivity of flagellin receptors, a simple downregulation of flagellin transcripts may not be sufficient to avoid recognition. Therefore, preventing the dissociation of flagella from the polymeric structure is a more plausible approach, as the majority of epitopes for flagellin receptors are located in the inner core channel of the flagella. The O-glycosylation of flagellin contributes to structural stability and suppresses the dissociation of flagellar filaments (Taguchi et al., 2009). Plants secrete enzymes such as β-galactosidase1 (BGAL1) that hydrolyze the glycosylation. Degradation of flagellin glycosylation by BGAL1 is required for FLS2-mediated immunity against *P. syringae* strains that carry terminal modified viosamine in their flagellin O-glycans, although the precise mechanism—whether involving promotion of flagellin monomer dissociation from filaments, facilitation of protease accessibility to generate immunogenic peptides, or both—remains to be determined (Buscaill et al., 2019). *P. syringae* pathovars counteract the role of BGAL1 through polymorphic glycans on flagellar structures or by producing a heat-stable small inhibitor molecule (<3 kDa) that remains to be identified (Buscaill et al., 2019).

### Camouflage

The polymorphism of epitope sequences in flagellin is also a strategy used by bacteria for immune evasion. The flg22 epitopes have been subject to strong evolutionary selection pressure. However, mutations in the address segment have a greater impact on bacterial motility than on immune evasion because FLS2 has a broad binding capacity for the address segment of different flg22 variants (Parys et al., 2021). Therefore, most immune-evading variants exhibit additional polymorphism in the message segment, which assists in antagonizing immunity through the perturbation of FLS2–BAK1 heterocomplex formation (Parys et al., 2021). For instance, *Ralstonia solanacearum* flg22 (flg22^Rso^) and *Agrobacterium tumefaciens* flg22 (flg22^Atum^) exhibit substantial divergence in the message segment compared with flg22 (Table 1) (Fürst et al., 2020; Wei et al., 2020). Although such camouflaged flagellins can evade FLS2 activation, some plant species have also evolved specific flagellin sensors to detect these flg22 peptides from their destructive pathogens. Soybean (*Glycine max*) has developed polymorphic FLS2 alleles and BAK1, which have evolved to recognize a broader spectrum of flg22, including flg22^Rso^ (Wei et al., 2020). Similarly, a grapevine species (*Vitis riparia*) employs an additional VrFLS2^XL^ allele that originated from a gene duplication event during the evolution of the *Vitis* lineage and recognizes both flg22 and flg22^Atum^ (Fürst et al., 2020). Thus, the plant host’s extensive allelic diversification of PRRs can counteract the ligand polymorphism that enables adaptive evasion of immunorecognition in pathogens (Vetter et al., 2012).

In natural communities isolated from healthy *Arabidopsis* plants, bacterial flg22 sequences can be categorized into three clades. Clade 1 (predominantly β- and γ-Proteobacteria FliCs) flg22 sequences exhibit a resemblance to flg22 and are mostly immunogenic. Clade 2 (predominantly Bacilli and Actinobacteria FliCs) flg22 sequences feature a slightly different address segment compared with that of flg22, resulting in partial immunogenicity. Clade 3 (predominantly Rhizobiales and Caulobacterales FliCs) flg22 sequences are the most divergent from flg22, particularly in the message segment. Clade 3 flg22 variants are mostly non-immunogenic, and a subset of non-immunogenic variants can compete with immunogenic flg22, leading to blocking of stable FLS2–BAK1 heterocomplex formation (Colaianni et al., 2021). Genomes with clade 3 *fliC* genes tend to maintain multiple *fliC* copies, implying that clade 3 communities prioritize immune evasion over flagellum-mediated motility for successful colonization and/or have reduced reliance on motility (Colaianni et al., 2021). In fact, bacteria with clade 3 flagellins become more dominant than bacteria with immunogenic flagellins when *Arabidopsis* is colonized by a synthetic community (SynCom), suggesting that microbes can also exploit FLS2-dependent defense responses to establish themselves in a particular niche (Colaianni et al., 2021). In nature, therefore, plant hosts collect traces from their surrounding microbiota community through the recognition of a mixed signature from flagellin by FLS2 and consequently fine-tune their defense responses to foster the assembly of specific commensal microbiota.

### Deception

#### Flagellin degradation/modification

FLS2-dependent defense responses are suppressed by numerous mechanisms that evolved independently in plant-associated bacteria (Teixeira et al., 2021). The plant-beneficial rhizobacteria *Pseudomonas* spp. suppress flg22-induced immune responses through production of gluconic acid to acidify the rhizosphere (Yu et al., 2019). Co-culturing *Arabidopsis* with SynComs results in suppression of flg22-induced root growth inhibition (Ma et al., 2021; Teixeira et al., 2021). Among the SynCom members, the *Dyella japonica* MF79 strain robustly suppresses flg22-induced defense responses through a proteinaceous molecule larger than 10 kDa, which is secreted via the type II secretion system (Teixeira et al., 2021). Since bacterial type I or II secretion systems secrete substrates into the extracellular environment, the proteinaceous molecule secreted by MF79 likely functions to suppress PTI in the apoplast. In addition, another SynCom member, *Janibacter* 101, uses a suppressive molecule larger than 3 kDa that is heat labile to suppress flg22-induced defense responses, possibly by modifying and/or degrading the flg22 peptide (Ma et al., 2021). In the context of community, these immunosuppressive members interfere with the plant immune response to facilitate community assembly, thus benefiting other non-suppressive members (Teixeira et al., 2021). The strategy of degrading flagellin epitopes is also commonly used by pathogenic bacteria. For instance, *P. aeruginosa* secretes an alkaline protease, AprA, that degrades monomeric flagellin through the type I secretion system to evade innate immune systems of both mammals and plants (Bardoel et al., 2011). AprA, whose expression and activity are regulated by the Las and Rhl quorum-sensing systems (Duan and Surette, 2007; Casilag et al., 2016), specifically targets flagellin monomers that are released during bacterial growth from flagellum construction or flagellar damage while leaving polymeric flagellin in intact flagella less affected. By specifically cleaving the conserved epitopes recognized by immune receptors, this protease enables the bacteria to maintain motility while effectively evading detection (Bardoel et al., 2011).

#### T3E-mediated direct interference with immune signaling

Pathogenic bacteria often operate a type III secretion system that injects type III effector proteins (T3Es) to disrupt plant defense responses inside host cells. Because T3Es are intensively studied and have been reviewed in previous publications, we will cover only T3Es that interfere with direct effectors of flagellin receptors in this review. The *P. syringae* effector AvrPto binds directly to FLS2 of *Arabidopsis* and tomato, blocking downstream signaling (Xiang et al., 2008). AvrPto prevents flg22-induced BIK1 phosphorylation, likely by blocking the autophosphorylation activity of FLS2; however, there is ongoing debate about whether AvrPto inhibits BAK1 or interferes with FLS2–BAK1 heterodimerization (Shan et al., 2008; Xiang et al., 2011). Another *P. syringae* effector, AvrPtoB, interacts with various PRRs, including FLS2, leading to degradation through polyubiquitination facilitated by its E3 ligase activity (Göhre et al., 2008). In addition, HopAO1 has been identified as an effector with tyrosine phosphatase activity that is produced in many *P. syringae* pathovars. It interacts with the FLS2 KD and decreases flg22-induced immune responses. Although tyrosine phosphorylation of EF-TU RECEPTOR (EFR), specifically at Tyr^836^, is known to be critical for its kinase activity, it remains unclear whether tyrosine phosphorylation is essential for FLS2 function (Macho et al., 2014).

## The emerging field of flagellin signaling

### PRR networking

Dynamic organization of PM lipids and proteins into a patchwork of membrane nanodomains that serve as dedicated platforms is inherently connected to various signaling pathways. FLS2 is heterogeneously distributed within the PM, providing evidence for the formation of nanodomain-localized receptor clusters (Bücherl et al., 2017). Indeed, PM receptors including FLS2 form a complex network of interactions, as illustrated by the cell-surface interaction network based on LRR motifs (Smakowska-Luzan et al., 2018). This networking can influence FLS2 signaling (Figure 3B). For example, FLS2-interacting receptor (FIR) and impaired oomycete susceptibility1 (IOS1) interact with both FLS2 and BAK1 receptors in a ligand-independent manner, positively regulating FLS2 signaling (Yeh et al., 2016; Smakowska-Luzan et al., 2018). By contrast, BAK1-interacting receptor-like kinase 2 (BIR2) and nuclear shuttle protein-interacting kinase 1 (NIK1) negatively regulate flg22-induced FLS2–BAK1 heterocomplex formation (Halter et al., 2014; Li et al., 2019). The abundance of FLS2 protein at the PM is also tightly regulated through its interacting receptors. QIAN SHOU KINASE (QSK1) negatively regulates FLS2-mediated PTI responses by promoting its vacuolar degradation through endocytosis or autophagy pathways (Goto et al., 2024). Furthermore, the stability and functionality of receptor networks, along with intricate interactions within membrane nanodomains, depend on the presence of key articulation-point receptors (Smakowska-Luzan et al., 2018). These receptors serve to connect multiple subnetworks, thereby playing a significant role in maintaining the overall integrity of the network. APEX stands out as a highly interactive node within the core LRR-RK network, implying its pivotal role alongside other articulation-point receptors in stabilizing the network. Further research is needed to precisely understand how receptor networks modulate FLS2 signaling through the control of membrane dynamics.

The tight regulation of FLS2 signaling by neighboring receptors is also observed with different types of receptors. The *Catharanthus roseus* receptor-like kinase 1-like (CrRLK1L) protein FERONIA (FER) senses cell wall integrity and is therefore involved in various signaling processes in plant physiology (Zhang et al., 2020). FER, along with its co-receptor LORELEI-like glycosylphosphatidylinositol-anchored protein 1 (LLG1), exhibits scaffolding functions in PTI signaling through interaction with FLS2 and BAK1 (Stegmann et al., 2017). By contrast, the CrRLK1L receptors ANXUR1/2 (ANX1/2) negatively regulate flg22-induced formation of the FLS2–BAK1 complex (Mang et al., 2017).

Clustering of PRRs and downstream signaling components within membrane nanodomains facilitates signaling processes, enhancing sensitivity to ligand molecules (Jaillais and Ott, 2019). Furthermore, dynamic rearrangements in response to different stimuli enable fine-tuning of receptor activation and signaling efficiency in a context-dependent manner. For example, specific lipid compositions within nanodomains, including sterols and phosphoinositides, can interact with scaffold proteins like remorins and flotillins (Jaillais and Ott, 2019; Legrand et al., 2019). In addition, myosin XIK acts as a molecular scaffold by interacting with remorin to recruit and stabilize BIK1 within FLS2-containing nanodomains, ensuring efficient immune-complex formation (Wang et al., 2024a). The interaction between scaffold proteins and these lipids contributes to the organization and stabilization of nanodomains, suggesting that receptor signaling is affected by different lipid compositions. This phenomenon can potentially explain tissue-specific differences in receptor activation or variations in receptor activation within the same cell along specific axes of cell polarity. Future investigations exploring the intricate interplay between the regulation of lipid composition within nanodomains and the responses of receptors could yield valuable insights into the mechanisms that govern plant immune responses.

### Phytocytokines

Receptor networking in FLS2 signaling is further modulated by phytocytokines, secreted peptides whose name is derived from metazoan cytokines because of their analogous roles in immune regulation. These peptides are recognized by their cognate PRRs. Among the best-characterized phytocytokines are the rapid alkalinization factors (RALFs), which are perceived by CrRLK1-family receptors such as the FER–LLG1 complex and regulate immune responses through modulation of receptor signaling networks (Stegmann et al., 2017; Xiao et al., 2019). In particular, RALF23 negatively regulates the flg22-induced FLS2–BAK1 heterocomplex formation promoted by FER and affects the membrane nanoscale organization of FLS2 and BAK1 (Stegmann et al., 2017; Gronnier et al., 2021). Mature RALF23 is produced in higher amounts by the subtilase site-1 protease (S1P) upon flg22 stimulation, suggesting a negative feedback mechanism in FLS2 signaling (Stegmann et al., 2017). A recent study demonstrated that RALF peptides and pectin fragments undergo liquid–liquid phase separation in the apoplast, resulting in the assembly of pectin–RALF–FER–LLG1 condensates, which induce indiscriminate receptor-cluster endocytosis (Liu et al., 2024). However, how these condensates engage in FLS2 signaling remains to be explained. In addition, ROOT MERISTEM GROWTH FACTOR 1 INSENSITIVE (RGI) receptors, which recognize the phytocytokine GOLVEN2/ROOT MERISTEM GROWTH FACTOR 9 (GLV2/RGF9), interact with FLS2 and contribute to its stabilization at the PM (Stegmann et al., 2022).

In addition to phytocytokine receptors that directly interact with FLS2, flg22 perception triggers the transcriptional upregulation of multiple phytocytokine families, establishing feedback loops that further modulate immune responses. Three notable examples include (1) pathogen-associated molecular pattern (PAMP)-induced peptides (PIPs), which amplify FLS2 signaling through the LRR-RK RLK7 (Hou et al., 2014); (2) SERINE-RICH ENDOGENOUS PEPTIDEs (SCOOPs), which enhance immune responses via perception by MALE DISCOVERER 1-INTERACTING RECEPTOR LIKE KINASE 2 (MIK2) and BAK1 (Hou et al., 2021; Rhodes et al., 2021); and (3) SCREW peptides, which are recognized by the LRR-RK NUT to regulate stomatal re-opening, reducing apoplastic water content and limiting pathogen proliferation (Liu et al., 2022). This coordinated upregulation of distinct phytocytokine families following flg22 perception illustrates how plants use transcriptional feedback mechanisms to fine-tune FLS2 signaling through diverse receptor pathways.

### Environmental effects

Although it is recognized that environmental factors can influence PTI responses, research on how environmental conditions affect microbial pathogenesis and plant immunity is still emerging. Understanding these environmental impacts on bacterial physiology and plant immunity could have broad significance for agricultural practices, enabling the optimization of plant defense strategies by integration of both factors (Choi and Lee, 2024). Elevated ambient temperatures (∼23°C–32°C) promote bacterial growth, increasing the likelihood of MAMP molecule production by pathogens. Accordingly, *Arabidopsis* PTI responses, including responses to flg22, are activated at these temperatures. By contrast, at lower ambient temperatures (∼10°C–23°C), reduced PTI responses and enhanced ETI responses occur owing to increased secretion of virulence effectors by pathogens (Cheng et al., 2013). However, other observations have been reported; longer exposure to elevated temperature (30°C) enhanced bacterial effector translocation into plant cells (Huot et al., 2017), and short-term heat stress (37°C and 42°C) suppressed PTI responses (Janda et al., 2019). The complex interplay between temperature, bacterial virulence, and plant immunity requires further thorough investigation within realistic climate contexts, as these varying experimental conditions poorly represent gradual, natural changes in temperature. Understanding how plants integrate temperature changes with their immune responses would provide more relevant agricultural insights. Blue-light intensity also influences PTI responses through the photoreceptor CRYPTOCHROME 1-mediated expression of LATE UPREGULATED IN RESPONSE TO HYALOPERONOSPORA PARASITICA (LURP1) protein, which undergoes N-terminal palmitoylation in response to flg22 and enhances FLS2 activity through increased interaction (Hao et al., 2024). Other abiotic factors, such as humidity and CO_2_ concentration, may also affect PTI responses because of their roles in stomatal immunity, although further studies on these interactions are required.

Nutrient availability in the soil can also influence PTI responses (Figure 3C). In general, nutrient deficiencies modulate plant immunity against rhizosphere bacteria, resulting in a root microbiome composition that differs from that in nutrient-rich soils. For example, low phosphate levels downregulate flg22-induced immune responses through the master transcriptional regulator PHR1 (Castrillo et al., 2017). PHR1 directly activates *RALF* expression under phosphate starvation, leading to reduced activation of PTI responses, including FLS2–BAK1 complex formation (Tang et al., 2022). Similarly, low iron availability leads to rhizosphere acidification for the promotion of iron solubility, and this can affect flg22-induced immune responses because FLS2 activity is dependent on apoplastic pH (Santi and Schmidt, 2009; Yu et al., 2019). A recent study identified crosstalk between flg22 and iron signaling, mediated by stabilization of systemic iron-deficiency signaling peptides known as iron mans (IMAs) (Platre et al., 2022; Cao et al., 2024). Moreover, high-density chemical-array interaction screening revealed that two synthetic Maya compounds can bind to FLS2 and act as weak agonists of FLS2 (Lee et al., 2024). In particular, Maya2 has a coumarin structure at its core. Given that coumarins are secreted by roots to facilitate iron acquisition and mediate the assembly of a beneficial root microbiome in iron-limiting soil environments (Harbort et al., 2020), coumarins may be involved in adjusting the optimization of FLS2 activity during plant–microbe interactions. In addition, the potential influence of photosynthesis, nitrogen fixation, and availability of other micronutrients on PTI responses is an intriguing area for future research. Investigating the impact of nutrient availability on plant immunity could provide new insights for optimizing plant defense mechanisms and improving agricultural practices.

## Concluding remarks

Over the past 5 years, significant questions in the flagellin-sensing pathway have been addressed, including the identification of flg22-processing proteases, the impact of flg22 sequence diversity on immune responses, species-specific variations in flagellin receptors, and the identification of previously unknown signal-transduction mechanisms (Fürst et al., 2020; Wei et al., 2020; Colaianni et al., 2021; Parys et al., 2021; Kong et al., 2024; Matsui et al., 2024; Karimi et al., 2025). Furthermore, recent synthetic biology approaches have expanded our understanding of FLS2 signaling, including the discovery of small-molecule surrogate ligands and the development of a rapamycin-inducible dimerization system (Kim et al., 2021; Lee et al., 2024). These approaches enable spatiotemporal control of receptor signaling, independent of cognate ligands. Although significant progress has been made in understanding the flagellin-sensing pathway, several questions remain unanswered or have emerged from recent advances. Key questions include (1) how do flagellin-sensing mechanisms simultaneously facilitate the colonization of commensal bacteria while restricting pathogenic bacteria in the rhizosphere and phyllosphere? (2) Do variations in the sensitivity and epitope preference of flagellin receptors among plant species contribute to shaping distinct microbiome compositions in their ecological context? (3) How do metabolites generated by plant-microbe or microbe–microbe interactions affect PTI responses, including flagellin sensing? (4) How does abiotic stress, especially climate change, influence flagellin sensing and signaling? (5) Why do flagellin-triggered immune responses exhibit a broader spectrum and higher amplitude than other MAMP/damage-associated molecular pattern-triggered responses, and which downstream signaling components contribute to this difference? (6) How do tissue- and cell-specific differences in flagellin perception and signaling contribute to the overall plant immune response and optimization of localized growth–immunity tradeoffs? (7) How can we apply our current understanding of flagellin-sensing mechanisms to improve crop disease resistance? To address these questions, a system-level approach—such as multi-omics that combines transcriptomics, proteomics, metabolomics, microbiome analysis, and metatranscriptomics—will be required. In addition, nanometer-scale super-resolution imaging or spatio-transcriptomics will provide valuable insights into cell-type-specific and cell-polarity-specific immune responses in plants. Technical advances in gene-editing tools and synthetic biology offer promising avenues for manipulating flagellin receptors to optimize plant responses, thereby providing tailored resistance against emerging pathogens and revolutionizing agricultural practices in an era of climate change. In this context, two promising ongoing studies involve the design of synthetic FLS2 receptors with broader flg22-variant recognition spectra through rational protein engineering, offering viable strategies for the development of crops with enhanced resistance to diverse pathogens (Li et al., 2024; Zhang et al., 2024).

As flagellin-sensing mechanisms are the most extensively studied in plant immunity, they will continue to serve as a key reference point, inspiring future research on other PRRs and their agricultural applications. These studies not only advance our understanding of plant immune responses but also offer valuable and feasible insights for driving future sustainable and resilient agriculture.

## Acknowledgments

We apologize to colleagues whose work could not be presented here owing to space limitations. We express our gratitude to all the members. This research was supported by grants from the 10.13039/501100010446Institute for Basic Science (IBS-R021-D1-2025-a00) and the 10.13039/501100003725National Research Foundation of Korea (RS-2024–00338015) to H.-S.L. and by the Startup Fund from 10.13039/501100015671Duke Kunshan University to E.Y.K. D.-H.L. was supported by a postdoctoral fellowship from the 10.13039/501100003725National Research Foundation of Korea (NRF-2021R1A6A3A03039464). No conflict of interest is declared.

## Author contributions

H.R., S.C., H.-S.L., and D.-H.L. wrote and edited the original draft. M.C., B.-K.K., and E.Y.K. reviewed and edited the article. H.R., S.C., and D.-H.L. designed the original draft figures. M.C., E.Y.K., H.-S.L., and D.-H.L. refined and finalized the figures. H.R. and S.C. prepared the table.
